# Seroprevalence Following the Second Wave of Pandemic 2009 H1N1 Influenza

**DOI:** 10.1371/currents.RRN1148

**Published:** 2010-04-25

**Authors:** Ted Ross, Shanta Zimmer, Don Burke, Corey Crevar, Donald Carter, James Stark, Brendan Giles, Richard Zimmerman, Stephen Ostroff, Bruce Lee

**Affiliations:** ^*^University of Pittsburgh; ^†^Department of Medicine, University of PIttsburgh School of Medicine; ^‡^the University of Pittsburgh; ^§^University of Pittsburgh, Pittsburgh Pa; ^#^The Center for Vaccine Research and ^††^Pennsylvania Department of Health

## Abstract

BACKGROUND: In April 2009, a new pandemic strain of influenza infected thousands of persons in Mexico and the United States and spread rapidly worldwide. During the ensuing summer months, cases ebbed in the Northern Hemisphere while the Southern Hemisphere experienced a typical influenza season dominated by the novel strain. In the fall, a second wave of pandemic H1N1 swept through the United States, peaking in most parts of the country by mid October and returning to baseline levels by early December. The objective was to determine the seroprevalence of antibodies against the pandemic 2009 H1N1 influenza strain by decade of birth among Pittsburgh-area residents. METHODS AND FINDINGS: Anonymous blood samples were obtained from clinical laboratories and categorized by decade of birth from 1920-2009. Using hemagglutination-inhibition assays, approximately 100 samples per decade (n= 846) were tested from blood samples drawn on hospital and clinic patients in mid-November and early December 2009. Age specific seroprevalences against pandemic H1N1 (A/California/7/2009) were measured and compared to seroprevalences against H1N1 strains that had previously circulated in the population in 2007, 1957, and 1918. (A/Brisbane/59/2007, A/Denver/1/1957, and A/South Carolina/1/1918). Stored serum samples from healthy, young adults from 2008 were used as a control group (n=100). Seroprevalences against pandemic 2009 H1N1 influenza varied by age group, with children age 10-19 years having the highest seroprevalence (45%), and persons age 70-79 years having the lowest (5%). The baseline seroprevalence among control samples from 18-24 year-olds was 6%. Overall seroprevalence against pandemic H1N1 across all age groups was approximately 21%. CONCLUSIONS: After the peak of the second wave of 2009 H1N1, HAI seroprevalence results suggest that 21% of persons in the Pittsburgh area had become infected and developed immunity. Extrapolating to the entire US population, we estimate that at least 63 million persons became infected in 2009. As was observed among clinical cases, this sero-epidemiological study revealed highest infection rates among school-age children.

## Background

In April of 2009, a new pandemic strain of influenza infected thousands of persons in Mexico and the United States and spread rapidly throughout the globe [1]. During the summer period, influenza occurred at low levels  in the Northern Hemisphere, whereas it dominated in the Southern Hemisphere [2].  In the fall, a second wave of pandemic H1N1 swept through the United States, peaking in most parts of the country by mid October and returning to baseline levels by early December. In Allegheny County (Pittsburgh), Pennsylvania the epidemic peaked in late October (personal communication, Kirsten Waller, Pennsylvania Department of Health, influenza surveillance data, 2009) in a largely unvaccinated community.  Estimates from Centers of Disease Control and Prevention (CDC) suggested that by December approximately 50 million people had been infected in the United States, however the true number of infected cases could not be measured with certainty due to a lack of serological evidence of asymptomatic cases. The epidemiology of pandemic H1N1 influenza appeared to be a mild to moderate disease affecting school-age children preferentially over older adults with elderly adults being underrepresented in severe cases [3] [4].  As with seasonal influenza, reporting of cases and hospitalizations underestimates the true infection rates in the population [4].  Asymptomatic and mild cases are missed by current reporting techniques and few studies have been performed to identify seroprevalence during an epidemic.  Although the pattern of disease preponderance among the young has been described historically with prior pandemic influenza [5] [6] [7] [8], characterization of serologic differences among various age groups with respect to various strains of influenza is lacking.  This study aimed to describe the community seroprevalence of antibodies to pandemic H1N1 at the time of the peak of the second pandemic wave and to characterize the existence of immunity to other historical strains of H1N1 influenza.  Measurement of the seroprevalence of H1N1 immunity provides valuable information about the likelihood of a possible third wave and may be useful in decision-making about immunization strategies.

## Methods:

### Sample cohorts and collections. 

The samples analyzed were excess serum samples collected anonymously from extra laboratory specimens from the University of Pittsburgh Medical Center’s Presbyterian Hospital and the Children’s Hospital of Pittsburgh from mid-November and early December 2009.  Pediatric samples were obtained from blood samples collected in outpatient clinics during the week of November 16, 2009.  Adult samples (older than 20 years) were obtained from the clinical laboratories of the UPMC hospitals during the week of November 23, 2009. University of Pittsburgh IRB approval [(exempt) #PRO09110164] was obtained.  Blood samples were collected using the honest broker system at the University of Pittsburgh Laboratories and given to investigators organized by decade of birth without other identifying information. Each serum sample was classified by decade of birth of the donor and tested in hemagglutination-inhibition assay (HAI) against pandemic H1N1 (A/California/7/2009), a seasonal H1N1 (A/Brisbane/59/2007) and 1918 H1N1 (A/South Carolina/1/1918).  A subset of samples (50% from each decade) were tested against and a historical H1N1 strain (A/Denver/1/1957) corresponding to the last H1N1 circulating prior to the 1957 pandemic of H2N2.  A set of serum samples (n=100) collected from young, healthy adults (average age 20.2 +/- 1.3 years) in 2008, prior to the pandemic (pre-pandemic), was used as controls for the assay.  Reference sera from individuals vaccinated with either inactivated trivalent seasonal Fluzone vaccine or pandemic H1N1 FluMist (GSK) vaccines were used as positive controls.  Using population data from Allegheny County (United States Census Bureau) [9], we extrapolated the number of people in each age group who are likely to be immune to pandemic H1N1 at the time of our study (Table 2).

### Generation of 1918 virus-like particles (VLPs).

HEK 293T cells (1x10^7^) were transfected using Lipofectamine2000 (Invitrogen) with 5mg of each plasmid DNA expressing A/South Carolina/1/1918 HA and A/Brevig Mission/1/1918 NA; and also 10mg of HIV-1_NL4-3_ *gag*. Cells were incubated for 72h at 37C and supernatants containing VLPs were harvested.  Supernatants were clarified by low speed centrifugation at 1000xg and sterile filtered using a 0.22mm filter.  VLPs were purified by centrifuging clarified supernatant at 100,000x*g* through a 20% glycerol cushion and resuspended in PBS.  Total protein was quantified via BCA protein assay (Pierce Chemical, Rockford, IL, USA) and VLPs were aliquoted and stored at -80C.

### Hemagglutination-inhibition (HAI) assays

Hemagglutination inhibition (HAI) assays were conducted as previously described [10] [11]. To inactivate non-specific inhibitors, aliquots of each serum sample were separately treated with receptor destroying enzyme (RDE) prior to being tested with a final serum dilution of 1:10 (starting dilution for the assays). Samples were serially diluted 2-fold into V-bottom 96-well microtiter plates. An equal volume of virus, adjusted to approximately 8HAunits/50μl was added to each well. The plates were covered and incubated at room temperature for 30 min followed by the addition of freshly prepared 1% turkey erythrocytes (RBCs) (Lampire Biologicals, Pipersville, PA, USA) in Phosphate buffered saline (PBS). The plates were mixed by agitation, covered, and allowed to set for 30 min at 25◦C.  The HAI titer was determined by the reciprocal of the last dilution which contained non-agglutinated RBCs. Positive and negative serum controls were included on each plate. Samples with HAI titers >1:40 were considered seropositive.

### Phylogeny of Hemagglutinin

Sequences were aligned with MUSCLE 3.7 software and the alignment was refined by Gblocks 0.91b software.  Phylogeny was determined using the maximum likelihood method with PhyML software and bootstrap values represent 100 cycles.  Trees were rendered using TreeDyn 198.3 software [12].  The NCBI accession numbers for the hemagglutinin (HA) sequences obtained through the Influenza Virus Resource [13][13] and used in phylogeny inference are as follows: ACP41105, A/California/04/2009; ACP41953, A/California/7/2009; ACQ99613, A/Mexico/4108/2009; ACA28844, A/Brisbane/59/2007; ABU50586, A/Solomon Islands/3/2006; ACD37430, A/New Caledonia/20/1999; ABD15258, A/Denver/1/1957; ABD77675, A/Puerto Rico/8/1934; AAD17229, A/South Carolina/1/1918; and AAD17219, A/New York/1/1918.

### Sample Size Calculations

Sample size calculations performed based on an estimated seroprevalence of 30% indicated that 89 samples would be required per decade to detect seroprevalence +/-10% within a 95% confidence interval.

### Statistical analysis 

Geometric mean HAI titers and standard error were calculated for each group. Sensitivity analysis was conducted around HAI titer cut offs of 1:40, 1:80 and 1:160 (data not shown) before the decision was made to use the conventional 1:40 as the cut off value for seropositivity.  To describe the cross-reactivity between various strains, Spearman’s correlation coefficients were calculated for each antigenic pair by decade.  Cochran-Armitage test for trend was calculated across age groups. Chi square tests were used to compare the difference in seropositivity between the seasonal and novel H1N1 strains for each decade.

## Results  

### Study population: 1920-2009

 In November and early December 2009, approximately 2-4 weeks after the peak of the fall wave in Allegheny County (Figure 1), serum samples were collected anonymously from 846 persons that ranged in age from 1 month to 90 years of age (Table 1).  Overall, ~21% of serum samples collected from all age groups were positive for the pandemic H1N1 influenza strain, A/California/7/2009.  This is in contrast to pre-pandemic samples from subjects in the 18-24 year-old age range, where only 6% of sera collected in 2008 were HAI positive for pandemic H1N1 (Table 1), likely due to cross-reactivity.  The percentage of persons with serum that tested positive for pandemic H1N1 influenza (A/California/7/2009) was highest among children in the 10-19 year old age group (46%) and the 0-9 year-old age group (29%).  The percentage of persons with serum that tested positive for HAI antibodies against pandemic H1N1 was 21% or less among the other age groups, with the lowest percentage of positive serum samples among individuals between 70-79 year olds (Table 1 and Figure 2).  However, the percentage of samples positive for pandemic H1N1 influenza was significantly higher in all age groups (p<0.05), except the 70 year olds, compared to pre-pandemic serum samples.  The test for trend demonstrated increasing seropositivity for both pandemic H1N1 among younger cohorts (p=0.001).  

**Figure:**
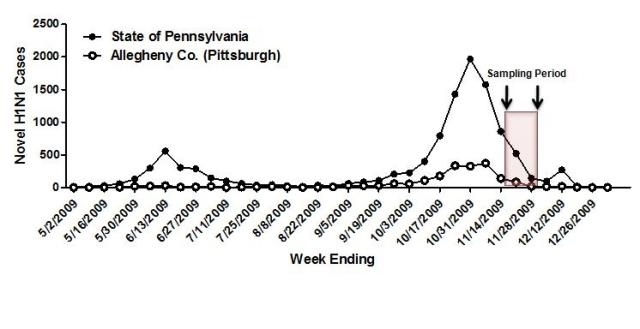
Figure 1. Allegheny County, Pennsylvania pandemic H1N1 cases. Sampling period for serosurvey (November 16-December 4, 2009) shown relative to epidemic curve. Distribution of the novel H1N1 vaccine to health clinics began in late-November.

**Figure:**
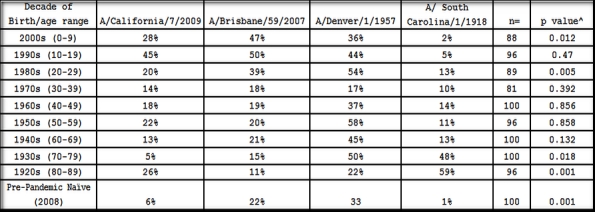

Table 1. Percentage of blood samples positive for influenza antibody by decade of birth and by selected influenza strains.

*(HAI>1:40) ^ Chi square testing the difference in seropositivity between A/California/7/2009 and A/Brisbane /59/2007; p<0.05 is statistically significant

Each serum sample was also tested for HAI activity against the seasonal H1N1 influenza strain, A/Brisbane/59/2007. This strain was the World Health Organization recommended H1N1 strain used by vaccine manufacturers for the past 3 influenza seasons [14] [15] [16]. Nearly 50% of serum samples from children younger than 20 years of age were positive for seasonal H1N1 and ~34% of serum samples from individuals 20-29 years of age. For all other age groups, 11-21% of serum samples were HAI positive for A/Brisbane/59/2007 (Table 1). The test for trend showed increasing seropositivity across the younger age groups (p<0.001). Serum samples collected from individuals in the 0-9, 20-29, 70-79, and 80-89 year old age groups had statistically (p<0.05) different percentage of positive samples against seasonal H1N1 influenza than pandemic H1N1 influenza (Table 1). Samples from each age group were also tested against historical strains of H1N1, A/Denver/1/1957 and A/South Carolina/1/1918. The percentage of samples positive for each of these strains by decade is described in Table 1 and depicted in Figure 2B.

**Figure:**
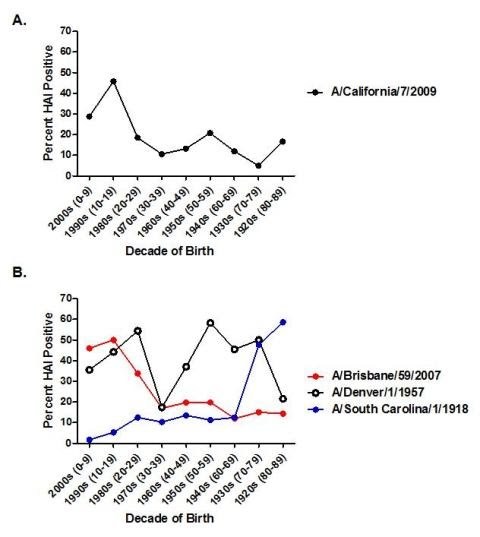

Figure 2.  Percent seropositive (HAI>1:40) by decade of birth for 4 strains of H1N1 influenza.A) Seropositive samples from pandemic H1N1 (A/California/7/2009).  B) Seropositive samples for historical influenza A H1N1 strains (A/Brisbane/59/2007, A/Denver/1/1957 and A/South Carolina/1/1918).  

To detect possible cross-reaction relationships between HAI titers among influenza strains, correlation coefficients were calculated for each decade and influenza strain tested. For each decade, the antigenic pair of A/California/7/2009 and A/South Carolina/1/1918 had stronger correlations than the antigenic pair of A/California/7/2009 and A/Brisbane/59/2007 and the pair of A/Brisbane/59/2007 and A/South Carolina/1/1918 except for the 1930-1939 decade.

We extrapolated the results from our sample to the local population (Table 2).  Thus, we estimate that 21.5% of the population in Allegheny County is seropositive to the novel 2009 H1N1 influenza, including over 70,000 school-age children. Extrapolating these results further to the entire US population, we estimate that 63 million persons became infected in 2009.

**Figure:**
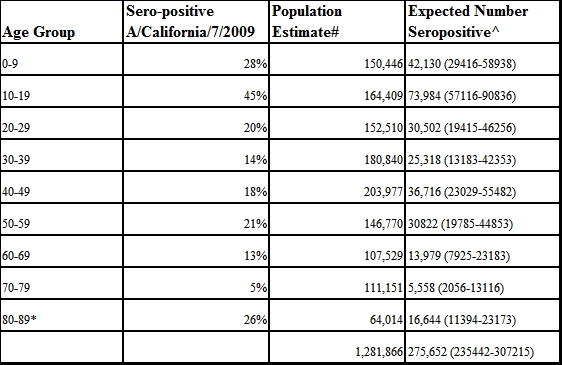

**Table 2. Expected population prevalence of immunity to pandemic H1N1 following peak of Second Wave in Allegheny County (Pittsburgh), PA.**

*Includes everyone over 85

# Population estimates taken from US Census (2000)

^ Estimated range based on 95% confidence limits of proportion sero-positive

**Table:** 

	**California/Brisbane positive**	**California/Denver positive**	**California/1918 positive**
**naïve **	3 of 7 (42%)	3 of 7 (42%)	0 of 7 (0%)
**1920**	9 of 24 (37.5%)	8 of 24 (33.3%)	19/24 (79.1%)
**1930**	0 of 5 (0%)	0 of 5 (0%)	1 of 5 (20%)
**1940**	4 of 13 (30.8%)	10 of 13 (76.9%)	7 of 13 (53.8%)
**1950**	3 of 20 (1.5%)	13 of 20 (65%)	14 of 20 (70%)
**1960**	7 of 17 (41.1%)	11 of 17 (64.7%)	13 of 17 (76.5%)
**1970**	5 of 11 (45.5%)	3 of 11 (27.3%)	10 of 11 (90.9%)
**1980**	11 of 18 (61.1%)	11 of 18 (61.1%)	12 of 18 (66.7%)
**1990**	29 of 42 (69%)	20 of 42 (47.6%)	17 of 42 (40.5%)
**2000**	14 of 24 (58.3%)	8 of 24 (33.3%)	5 of 24 (20.8%)
	**CA/Bris/Den positive**	**CA/Bris/1918 positive**	**CA/Den/1918 positive**
**naïve **	2 (28.6%)	0	0
**1920**	0	1 (4.2%)	1 (4.2%)
**1930**	0	0	0
**1940**	3 (23.1%)	0	3 (23.1%)
**1950**	0	0	4 (20%)
**1960**	0	0	2 (11.8%)
**1970**	0	3 (27.3%)	0
**1980**	3 (16.7%)	1 (5.6%)	0
**1990**	8 (19%)	2 (4.8%)	1 (2.4%)
**2000**	4 (16.7%)	0	0
	**CA/Bris/Den/1918 positive**
**naïve **	0
**1920**	5 (20.9%)
**1930**	0
**1940**	1 (7.7%)
**1950**	2 (10%)
**1960**	6 (35.3%)
**1970**	2 (18.2%)
**1980**	7 (38.9%)
**1990**	8 (19%)
**2000**	3 (12.5%)

## Discussion

The emergence of a novel H1N1 strain challenged public health officials and scientists to make prompt decisions about how best to prevent transmission in the face of an imminent pandemic. The need for more comprehensive serosurveys to understand infection rates and population immunity has emerged as a significant tool missing from our armamentarium.  This study, which examined real-time seroprevalence shortly after the fall wave (considered the second pandemic wave) in the United States, contributes to our understanding of the spread of the pandemic through the population. It also sheds light on the hypothesis that prior exposure to H1N1 influenza contributes to population immunity against this novel strain and may explain some of the differential distribution of affected age groups. A population based, prospective serologic study performed by the Health Protection Agency (HPA) earlier this year in the UK contributed to the questions of population immunity and incidence of this disease in distinct groups in the United Kingdom (UK) [17]. While providing information on incident cases, the UK study also highlighted important geographic differences in spread of the disease even within the same country and raised questions about the mechanism for relative protection among the elderly.  The UK finding of 31% baseline seroprevalence among elderly (greater than 80 years old) [17] suggests a cause for decreased infection in this population and is supported by the findings of our study which show high reaction to A/South Carolina/1/1918 among subjects born in the 1920s with a high correlation between seropositivity to the pandemic H1N1 and the 1918 strain.  Using microneutralization assays, a CDC evaluation of cross-protective antibodies among prior seasonal influenza vaccinees demonstrated very low pre-existing antibodies among the young with 33% cross-reactivity among people over 60 years [18].  Similarly, a studies of stored serum samples from the pre-pandemic period found some level of cross-reactive antibody in older adults [19] and that the oldest individuals (born between 1909-1919; age 90-100) had antibodies against the 2009 novel H1N1 influenza virus [20], consistent with prior infection with 1918 influenza. In contrast, most individuals born after 1944 lacked antibodies to the novel H1N1-like viruses.  The 1918-like influenza viruses and the 2009 novel H1N1-like viruses are genetically related to the pandemic H1N1 strain (Figure 3) through the classic swine influenza lineage [21] [22] and based on the three-dimensional structure of the HA molecule, the antigenic epitopes of the novel 2009 H1N1 HA are more closely related to those of the 1918-like influenza HA molecules than to those of contemporary seasonal H1N1 influenza viruses [20].

**Figure:**
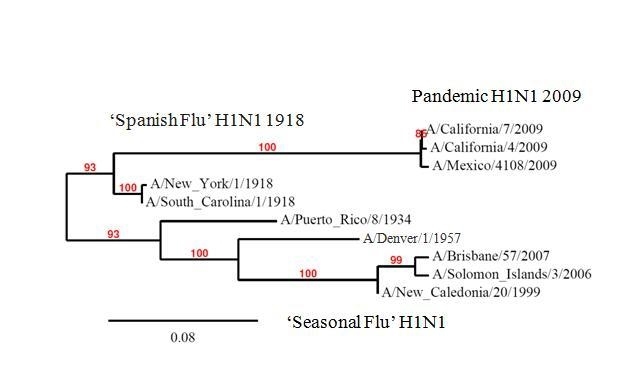
**Figure 3. Genetic relatedness of HA from historical strains of influenza H1N1.**  The tree includes select H1N1 influenza isolates of the classic swine and human influenza lineages (see Materials and Methods for database accession numbers).  Phylogenetic trees were inferred from hemagglutinin amino acid sequences using the maximum likelihood method.  Bootstrap analysis values are shown above the branches.  The scale bar indicates the number of amino acid residue changes per unit length of the horizontal branches.

Building on the work of Robert Shope in the 1930s [23], Thomas Francis, Jr. coined the term “original antigenic sin” in 1955 [24] [25] to describe the observation that the antibody response to the first influenza infection of childhood remains the dominant antibody response thoughout life. This hypothesis, originally based on studies of human antibody response to various strains of influenza viruses, was further supported by sequential experimental infections in ferrets [26] [27] and has been an important concept in the understanding of influenza immunology and a challenge for influenza vaccine development [28] [29].

The patterns of antibodies against historical H1N1 strains seen in this study (Figure 2) demonstrate expected age-specific differences in prior influenza A infections based on known circulation of H1N1 in the past century. In the elderly, the combination of  decreased disease pandemic influenza incidence, the high levels of pre-existing antibodies against novel H1N1, and our finding of the high seroprevalence of antibodies to the 1918 influenza strain, support the hypothesis of a role for the long lasting immunologic memory of the initial influenza infection.  Correlations between the HAI antibody titers against 2009 pandemic H1N1 and the 1918 pandemic strain in individual sera are also supportive. In contrast, seasonal H1N1 HAI results showed only weak correlations with pandemic H1N1, especially the 1957 Denver strain.  The finding of 26% seroprevalence of antibodies against 2009 pandemic H1N1 among those born in the 1920s may represent both cross-reactivity with the assay and evidence for cross-protective immunity.  The immunity afforded by the presence of 57% seroprevalence of antibodies against the 1918 H1N1 could explain the lower incidence of pandemic H1N1 infection seen among the elderly in the pandemic thus far.    

In school age children, intense social mixing patterns are known to drive transmission of respiratory viruses, especially influenza [30].  Our results suggest that absence of pre-existing immunity to the new strain, especially from early historical H1N1 strains (1918, 1957), rendered children susceptible to the emerging virus, and close socialcontact patterns further enhanced rapid spread of the virus. The milder disease seen with this pandemic may also have contributed to increased spread as more asymptomatic or minimally symptomatic students continued to circulate among their peers. Our results showing high seroprevalence of antibodies against novel H1N1 (45%) and low cross reactive antibodies against the most highly genetically related 1918 strain (5%) among children 10- 20 years old are supportive of both immune-related and contact-related susceptibility.  Further support of the role of social mixing in the disease spread comes from more detailed sub-analysis of the age-specific immunity pattens of the children less than 10 years old.  Those in the school age group (5-9 year olds) were 50% positive, while very few samples from those less than 5 years old were positive (data not shown).  Further work to correlate epidemiologic data and immunologic responses to hemagglutinin from different interpandemic periods should be done.

Production of a timely study that was consistent with US privacy regulations in the setting of a rapidly evolving pandemic proved difficult, and we faced several limitations. Because we used anonymous laboratory specimens, clinical information such as history of influenza-like illness, or vaccination status was unavailable. However, the timing of the sampling relative to vaccine availability in Pittsburgh suggests that these samples are likely from a largely unvaccinated population during the peak of the second pandemic wave.  While the pediatric samples were mostly outpatient samples, the adult samples arise from a hospitalized cohort and therefore may under-represent true seropositivity among community dwelling healthy adults who might be expected to engage in more social mixing than hospitalized adults. This would be especially true of the young, working age adults in their 30s to 50s (decades of birth 1960s and 1970s).  However, given the similarity between these results and published epidemiology of the pandemic thus far, the sample appears to be fairly representative of what has been seen throughout the globe. It is important to note that samples were collected approximately 2-3 weeks after the pandemic peak implying that seroprevalence in all age groups is likely to be higher at this point in time, allowing for further seroconversion from both natural infection and vaccination. Finally, the specimens were a convenience sample from excess blood at participating clinical laboratories, and not a true random sampling of the population. 

## Conclusion

The novel 2009 H1N1 influenza strain has been found to have a relatively low transmissibility, *i.e.* an R_0_ [31] [32] of 1.3. Our finding of high anti-2009 H1N1seroprevalences among school children and high anti-1918 H1N1 seroprevalences among the elderly suggest that further sustained viral transmission  is not likely.  With current estimates of seroprevalence and continued increases in population due to vaccination, a significant change in viral antigens or a change in population immunity would be required for further disease spread.  However, we cannot rule out the possibility that geographical pockets of limited immunity may be present in which a third wave may yet occur.  Ongoing viral and serosurveillance efforts will be essential to inform decisions around vaccination and other disease mitigating strategies.

## Acknowledgements

The authors would like to thank the staff of the laboratories of the University of Pittsburgh Medical Center for their assistance with sample acquisition, and Steve Wisniewski for initial sample size calculations.  We thank Elodie Ghedin for providing helpful comments.  

## Funding Information

This study was funded by NIH/NIAID supplemental A.R.R.A. to U01AI077771 to T.M.R. and a research pilot award from the University of Pittsburgh, Graduate School of Public Health (D.S.B and S.M.Z.).

## Competing Interests

The authors have declared that no competing interests exist.

## References

[ref-871418571] Dawood FS, Jain S, Finelli L, Shaw MW, Lindstrom S, et al. (2009) Emergence of a novel swine-origin influenza A (H1N1) virus in humans. N Engl J Med 360: 2605-2615 10.1056/NEJMoa0903810 19423869

[ref-3579478879] Bishop JF, Murnane MP, Owen R (2009) Australia's winter with the 2009 pandemic influenza A (H1N1) virus. N Engl J Med 361: 2591-2594. 10.1056/NEJMp0910445 19940287

[ref-623687342] Simonsen L, Clarke MJ, Schonberger LB, Arden NH, Cox NJ, et al. (1998) Pandemic versus epidemic influenza mortality: a pattern of changing age distribution. J Infect Dis 178: 53-60. 10.1086/515616 9652423

[ref-75690171] Reed C, Angulo FJ, Swerdlow DL, Lipsitch M, Meltzer MI, et al. (2009) Estimates of the prevalence of pandemic (H1N1) 2009, United States, April-July 2009. Emerg Infect Dis 15: 2004-2007. 10.3201/eid1512.091413 PMC3375879 19961687

[ref-187709759] Webster RG (1972) On the origin of pandemic influenza viruses. Curr Top Microbiol Immunol 59: 75-105. 10.1007/978-3-642-65444-2_3 4570690

[ref-99993769] Langmuir AD, Schoenbaum SC (1976) The epidemiology of influenza. Hosp Pract 11: 49-56. 10.1080/21548331.1976.11707011 67988

[ref-1620218789] Rao BL (2003) Epidemiology and control of influenza. Natl Med J India 16: 143-149. 12929857

[ref-1157742224] Kilbourne ED (2006) Influenza pandemics of the 20th century. Emerg Infect Dis 12: 9-14. 10.3201/eid1201.051254 PMC3291411 16494710

[ref-2078399870] U.S. Census Bureau Census 2000, Summary File 1.

[ref-3448103517] Bright RA, Carter DM, Crevar CJ, Toapanta FR, Steckbeck JD, et al. (2008) Cross-clade protective immune responses to influenza viruses with H5N1 HA and NA elicited by an influenza virus-like particle. PLoS One 3: e1501. 10.1371/journal.pone.0001501 PMC2200794 18231588

[ref-1127513108] Bright RA, Carter DM, Daniluk S, Toapanta FR, Ahmad A, et al. (2007) Influenza virus-like particles elicit broader immune responses than whole virion inactivated influenza virus or recombinant hemagglutinin. Vaccine 25: 3871-3878. 10.1016/j.vaccine.2007.01.106 17337102

[ref-3906067565] Dereeper A, Guignon V, Blanc G, Audic S, Buffet S, et al. (2008) Phylogeny.fr: robust phylogenetic analysis for the non-specialist. Nucleic Acids Res 36: W465-469. 10.1093/nar/gkn180 PMC2447785 18424797

[ref-2583761268] Bao Y, Bolotov P, Dernovoy D, Kiryutin B, Zaslavsky L, et al. (2008) The influenza virus resource at the National Center for Biotechnology Information. J Virol 82: 596-601. 10.1128/JVI.02005-07 PMC2224563 17942553

[ref-3657482919] Fiore AE, Shay DK, Haber P, Iskander JK, Uyeki TM, et al. (2007) Prevention and control of influenza. Recommendations of the Advisory Committee on Immunization Practices (ACIP), 2007. MMWR Recomm Rep 56: 1-54. 17625497

[ref-975631067] (1998) Recommendations for prevention and control of hepatitis C virus (HCV) infection and HCV-related chronic disease. Centers for Disease Control and Prevention. MMWR Recomm Rep 47: 1-39. 9790221

[ref-4002985672] (2009) Use of influenza A (H1N1) 2009 monovalent vaccine: recommendations of the Advisory Committee on Immunization Practices (ACIP), 2009. MMWR Recomm Rep 58: 1-8. 19713882

[ref-85884222] Miller E, Hoschler K, Hardelid P, Stanford E, Andrews N, et al. (2010) Incidence of 2009 pandemic influenza A H1N1 infection in England: a cross-sectional serological study. Lancet. In press. 10.1016/S0140-6736(09)62126-7 20096450

[ref-1579730135] (2009) Serum cross-reactive antibody response to a novel influenza A (H1N1) virus after vaccination with seasonal influenza vaccine. MMWR Morb Mortal Wkly Rep 58: 521-524. 19478718

[ref-3790199517] Hancock K, Veguilla V, Lu X, Zhong W, Butler EN, et al. (2009) Cross-Reactive Antibody Responses to the 2009 Pandemic H1N1 Influenza Virus. N Engl J Med. 361:1945-52. 10.1056/NEJMoa0906453 19745214

[ref-3779179918] Ikonen N, Strengell M, Kinnunen L, Österlund P, Pirhonen J, et al. (2010) High frequency of cross-reacting antibodies against 2009 pandemic influenza A(H1N1) virus among the elderly in Finland. Euro Surveill 15: 19478. 20144443

[ref-2496925128] Shinde V, Bridges CB, Uyeki TM, Shu B, Balish A, et al. (2009) Triple-reassortant swine influenza A (H1) in humans in the United States, 2005-2009. N Engl J Med 360: 2616-2625. 10.1056/NEJMoa0903812 19423871

[ref-3300771445] Garten RJ, Davis CT, Russell CA, Shu B, Lindstrom S, et al. (2009) Antigenic and genetic characteristics of swine-origin 2009 A(H1N1) influenza viruses circulating in humans. Science 325: 197-201. 10.1126/science.1176225 PMC3250984 19465683

[ref-1620399294] Shope RE (1936) The Incidence of Neutralizing Antibodies for Swine Influenza Virus in the Sera of Human Beings of Different Ages. J Exp Med 63: 669-684. 10.1084/jem.63.5.669 PMC2133359 19870496

[ref-85360366] Francis T, Jr. (1955) The current status of the control of influenza. Ann Intern Med 43: 534-538. 10.7326/0003-4819-43-3-534 13249235

[ref-3125885825] Francis T, Jr. (1960) On the Doctrine of Original Antigenic Sin. Proceedings of the American Philosophical Society 104: 572-578.

[ref-2647069377] Jensen KE, Davenport FM, Hennessy AV, Francis T, Jr. (1956) Characterization of influenza antibodies by serum absorption. J Exp Med 104: 199-209. 10.1084/jem.104.2.199 PMC2136660 13345965

[ref-2354310685] Webster RG (1966) Original antigenic sin in ferrets: the response to sequential infections with influenza viruses. J Immunol 97: 177-183. 5921310

[ref-1437403640] Kim JH, Skountzou I, Compans R, Jacob J (2009) Original antigenic sin responses to influenza viruses. J Immunol 183: 3294-3301. 10.4049/jimmunol.0900398 PMC4460008 19648276

[ref-3820593274] Carrat F, Flahault A (2007) Influenza vaccine: the challenge of antigenic drift. Vaccine 25: 6852-6862. 10.1016/j.vaccine.2007.07.027 17719149

[ref-2185239849] Mossong J, Hens N, Jit M, Beutels P, Auranen K, et al. (2008) Social contacts and mixing patterns relevant to the spread of infectious diseases. PLoS Med 5: e74. 10.1371/journal.pmed.0050074 PMC2270306 18366252

[ref-4236517209] Yang Y, Sugimoto JD, Halloran ME, Basta NE, Chao DL, et al. (2009) The transmissibility and control of pandemic influenza A (H1N1) virus. Science 326: 729-733. 10.1126/science.1177373 PMC2880578 19745114

[ref-1268089001] Fraser C, Donnelly CA, Cauchemez S, Hanage WP, Van Kerkhove MD, et al. (2009) Pandemic Potential of a Strain of Influenza A (H1N1): Early Findings. Science 324: 1557-1561. 10.1126/science.1176062 PMC3735127 19433588

